# Peripheral T-Cell Lymphoma with Aberrant Expression of CD19, CD20, and CD79a: Case Report and Literature Review

**DOI:** 10.1155/2013/183134

**Published:** 2013-08-28

**Authors:** Rahul G. Matnani, Rachel L. Stewart, Joseph Pulliam, Chester D. Jennings, Melissa Kesler

**Affiliations:** Department of Pathology & Laboratory Medicine, Chandler Medical Center, College of Medicine, University of Kentucky, 800 Rose Street, Lexington, KY 40536-0298, USA

## Abstract

A case of lymphoma of T-cell derivation with aberrant expression of three B-cell lineage markers (CD19, CD20, and CD79a), which was diagnosed on a left axillary excision, is described. Immunohistochemical studies and flow cytometry analysis demonstrated neoplastic cells expressing CD3, CD19, CD20, and CD79a with absence of CD4, CD8, CD10, CD30, CD34, CD56, CD68, TDT, MPO, PAX-5, and surface immunoglobulin. Gene rearrangement studies performed on paraffin blocks demonstrated monoclonal T-cell receptor gamma chain rearrangement with no evidence of clonal heavy chain rearrangement. The neoplastic cells were negative for Epstein-Barr virus (EBV) or Human Herpes Virus 8 (HHV-8). At the time of diagnosis, the PET scan demonstrated hypermetabolic neoplastic cells involving the left axilla, bilateral internal jugular areas, mediastinum, right hilum, bilateral lungs, and spleen. However, bone marrow biopsy performed for hemolytic anemia revealed normocellular bone marrow with trilineage maturation. The patient had no evidence of immunodeficiency or infection with EBV or HHV-8. This is the first reported case of a mature T-cell lymphoma with aberrant expression of three B-cell lineage markers. The current report also highlights the need for molecular gene rearrangement studies to determine the precise lineage of ambiguous neoplastic clones.

## 1. Introduction

In vast majority of cases, T-cell lymphomas do not express B-cell markers. However, aberrant expression of one or two B-cell antigens has been documented. However, there are no cases reported in the literature of peripheral T-cell lymphomas expressing three B-cell immunophenotypic markers. Here, we present a case of a gamma clonal peripheral T-cell lymphoma expressing CD19, CD20, and CD79a in an individual with no evidence of immunodeficiency or infection with EBV or HHV-8.

CD19 is a transmembrane protein that is expressed on B cells and follicular dendritic cells. CD19 along with CD21 plays an important role in B-cell activation. Aberrant expression of CD19 has been reported in acute myeloid leukemia [1, 2]. However, only one case report has described aberrant expression of only CD19 in mature T-cell lymphoma [3]. 

CD20 is a B-cell lineage-specific marker expressed on the surface of all B cells, except the early pro-B cells and terminal plasma cells. This 37 kDa nonglycosylated phosphoprotein may act as calcium channel in the cell membrane [4]. Although the exact role of CD20 and its ligand is still unclear, CD20 is believed to boost T-cell-independent antibody response [5]. CD20 expression is diagnostic of B-cell lymphomas, hairy cell leukemia, melanoma, thymoma, and Hodgkin's disease. Monoclonal antibodies such as rituximab and tositumomab target the expression of CD20 on B-cell leukemia and lymphoma [6, 7]. However, few case reports demonstrating expression of CD20 in peripheral T-cell lymphomas have been reported [8–13]. 

CD79a (mb-1) protein is associated with IgM. Due to its association with the B-cell antigen receptor, it is expressed exclusively on the B cells and is absent in other healthy immune cells. However, its aberrant expression has been reported in both B- and T-cell lymphoblastic leukemia [14–17]. However, similar to the current case report, only one case report described CD79a expression in a mature T-cell lymphoma [18]. Interestingly, Yao et al. also reported an aberrant expression of CD20 along with CD79a in the gamma clonal T-cell lymphoma. To our knowledge, this is the first case report of a peripheral mature T cell lymphoma expressing CD19, CD20, and CD79a B cell markers.

## 2. Case Presentation

The patient was a 75-year-old Caucasian male who underwent a left axillary dissection for enlarging lymph nodes present for more than one year. He denied symptoms of fatigue, dyspnea, fever, or night sweats. The PET scan performed at the same time demonstrated hypermetabolic foci involving the left axilla, bilateral internal jugular areas, mediastinum, right hilum, bilateral lungs, and spleen. Interestingly, a year ago, he had an episode of hemolytic anemia for which he completed a one-year course of oral prednisone. Further evaluation of clinical history revealed that he was diagnosed two years back with reactive cervical lymph node at an outside hospital. Moreover, five years before the current diagnosis of T-cell lymphoma, the patient also had sinus histiocytosis involving level 7 neck lymph node. His past history was significant for vocal cord resection for tumor more than 40 years ago. His social history was significant for exposure to asbestos as a construction worker. His father and two uncles died of prostate cancer. There was no family history of hematological malignancies or blood cell dyscrasias.

**Figure 1:**
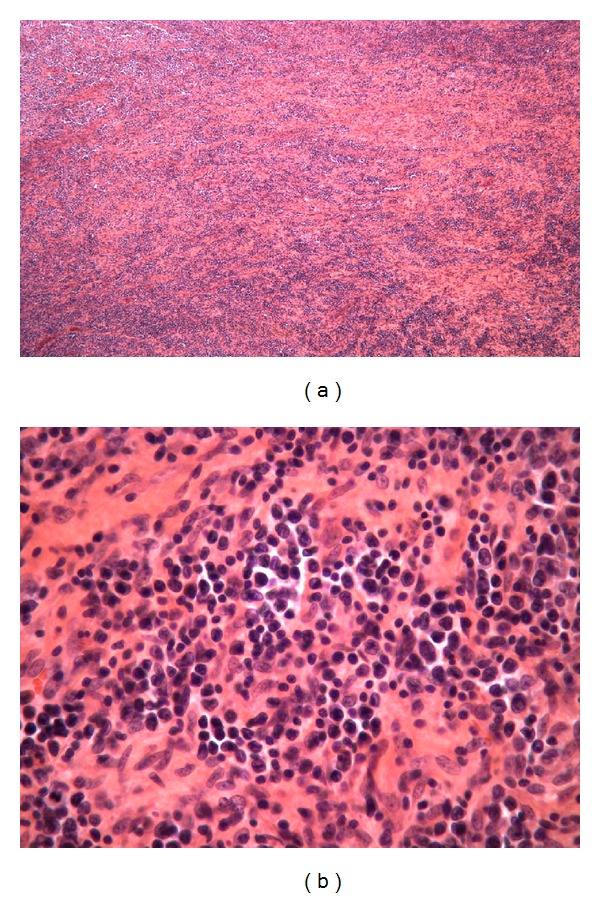
(a) Atypical lymphoid infiltrates associated with marked fibrosis (20x magnification) and (b) atypical cells with irregular nuclear membrane, open chromatin, and distinct nucleoli (200x magnification).

**Figure 2:**
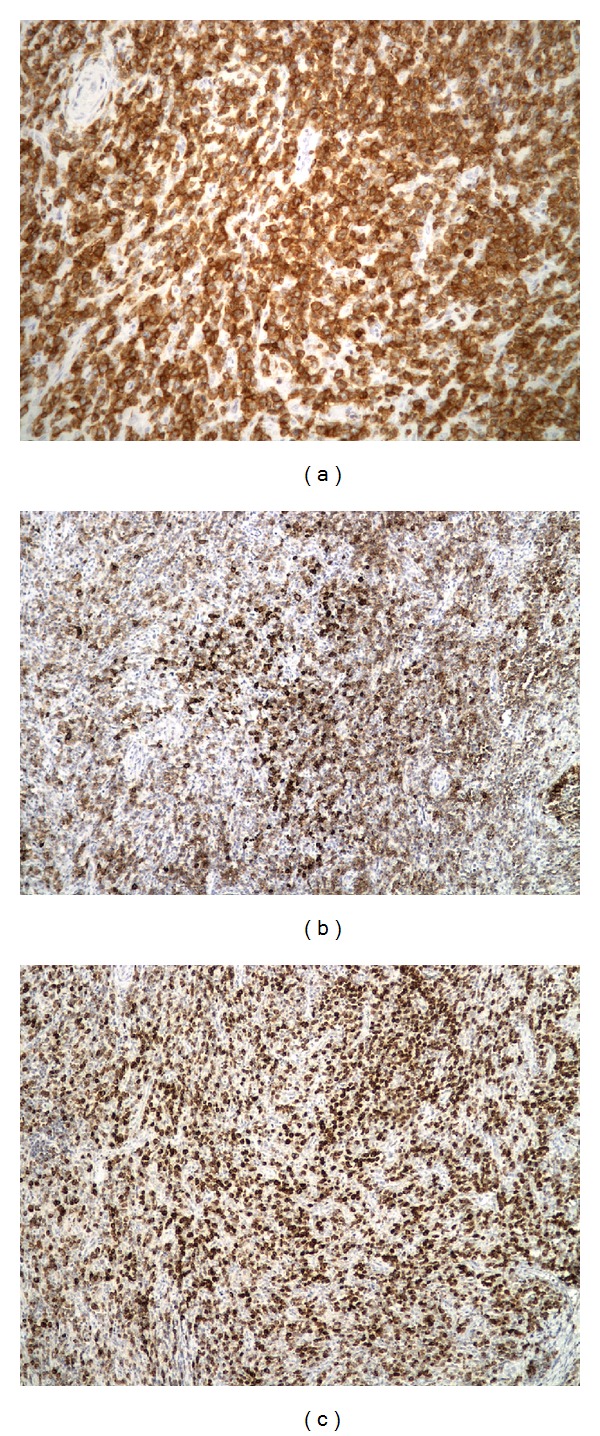
(a) Neoplastic cells demonstrating strong CD3 positivity (200x magnification), (b) neoplastic T cells with aberrant CD20 positivity (100x magnification), and (c) neoplastic T cells with aberrant CD79a positivity (100x magnification).

**Figure 3:**
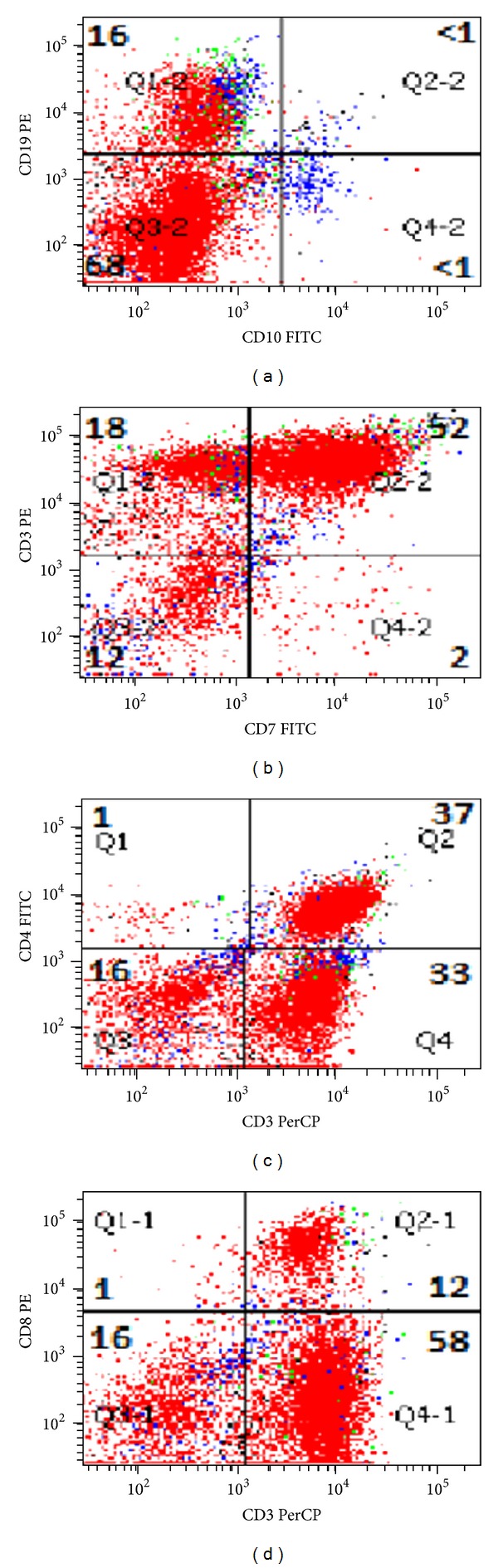
Flow cytometry demonstrating aberrant T cells expressing CD3 and CD19, weak CD7, and absent CD4, CD8, and CD10 expression.

The histological sections demonstrated an effaced nodal architecture with proliferation of large atypical lymphoid infiltrates associated with marked fibrosis (Figure 1(a)). The atypical cells showed irregular nuclear membrane, open chromatin, and distinct nucleoli (Figure 1(b)). The neoplastic cells stained strongly and diffusely positively for CD3 with partial loss of CD5 and CD7 (Figure 2(a)). A subpopulation of the neoplastic cells was positive for CD20 and CD79a (Figures 2(b) and 2(c)). The neoplastic cells were negative by immunohistochemistry for CD4, CD8, CD10, CD30, CD34, CD56, CD68, TDT, MPO, PAX-5, EBV, and HHV8. Further review of flow cytometry histograms demonstrated an aberrant T-cell population, which expressed CD19 along with CD3 and CD20 (Figure 3). The gene rearrangement studies for immunoglobulin and T-cell receptors were performed by ARUP laboratories Inc., Salt lake City, Utah, by performing polymerase chain reaction on the DNA extracted from paraffin embedded blocks. The T-cell clonality was confirmed by gene rearrangement studies showing gamma clonal T-cell receptor rearrangement with absence of immunoglobulin chain gene rearrangement.

## 3. Discussion

The current case report demonstrates the need to utilize genetic rearrangement studies to determine the precise lineage for neoplastic clones expressing biphenotypic markers by flow cytometry and immunohistochemistry. It is possible to misdiagnose the current case as a large B-cell lymphoma expressing CD19, CD20, and CD79a. Interestingly, five years prior to the diagnosis of peripheral T-cell lymphoma, the patient had a previous diagnosis of sinus histiocytosis on a level 7 lymph node excision. In addition, three years later, he was diagnosed with a reactive cervical lymph node at an outside hospital. Unfortunately, at the time of diagnosis, the PET scan demonstrated hypermetabolic neoplastic cells involving the left axilla, bilateral internal jugular areas, mediastinum, right hilum, bilateral lungs, and spleen. However, bone marrow biopsy performed for hemolytic anemia revealed normocellular bone marrow with trilineage maturation. 

The current case demonstrates an unusual occurrence of genetic dysregulation involving three B-cell specific lineage markers leading to lineage infidelity. This genetic dysregulation may involve transcription factors regulating the expression of B-cell markers. Recently, it has been demonstrated that PAX-5 induces transcriptional activation of CD19 and CD79a in B cells [19]. However, in our case, the T-cell neoplastic clone did not express PAX-5, by immunohistochemistry. In addition, lineage infidelity may be demonstrated in the presence of immunodeficiency and infection with EBV or HHV-8 [20–22]. In the current case, the patient did not show any evidence of immunodeficiency. Immunohistochemistry of the neoplastic cells was negative for EBV and HHV-8.

The findings in our case suggest a T-cell lymphoma belonging to the *γδ* T-cell lineage. Interestingly, the tumor was positive for CD3 but was negative for CD56 that is expressed on *γδ* T-cell lymphomas [23, 24]. Based on the involvement of tissues by neoplastic cells by PET scan, the neoplastic clone reported in our study showed an aggressive clinical behavior. However, the current patient showed an excellent response to six cycles of CHOP chemotherapy regimen along with Rituxan. Unfortunately, a follow-up CT scan three months later showed progression of lung nodules. He subsequently underwent salvage chemotherapy with three cycles of ICE chemotherapy regimen. A follow-up CT scan showed complete response after three cycles of ICE chemotherapy with no evidence of pulmonary nodules. In addition, his bone marrow biopsy showed no evidence of involvement by neoplastic cells.

Thus, the current study demonstrates that the use of more than one B-cell marker by flow cytometry or immunohistochemistry may not be able to precisely identify the lineage of neoplastic clones. In these cases of lineage infidelity, genetic rearrangement studies are invaluable for providing a conclusive answer. Moreover, the aberrant expression of three B-cell markers in a peripheral T-cell lymphoma suggests a high degree of transcriptional dysregulation of genes, which may be closely linked to initiation and/or proliferation of the neoplastic clone.
